# SF method for removing small skin melanocytic nevus

**DOI:** 10.3389/fsurg.2024.1451660

**Published:** 2024-08-09

**Authors:** Jianfei Zhang, Kaixi Tan, Yujun Tang, Xia Xiao, Feng Yang, Jie Chen

**Affiliations:** ^1^ Department of Burns and Plastic Surgery, The Second Affiliated Hospital, Hengyang Medical School, University of South China, Hengyang, China; ^2^ Department of Burns and Plastic Surgery, The Affiliated Nanhua Hospital, Hengyang Medical School, University of South China, Hengyang, China

**Keywords:** SF method, skin surgery, melanocytic nevus, SCAR, surgical technique

## Abstract

**Background:**

The most prevalent benign skin tumor is a melanocytic nevus, which can potentially turn cancerous and frequently impair a patient's appearance.

**Objective:**

To find a standardized surgical procedure for the removal of tiny skin pigmented nevis and to look into the effectiveness of the SF technique for doing so.

**Methods:**

A retrospective study was conducted on 723 patients with small-sized pigmented nevi between June 2022 and June 2023. The nevi were removed using the SF method, and the patients' overall health and the duration of the procedure were recorded. Three months following the procedure, the patients completed a questionnaire to assess the effectiveness, recurrence, complications, and degree of satisfaction with the surgical outcome.

**Results:**

Compared with the normal method, SF method had shorter operation time, higher effective rate, no recurrence and other postoperative complications after 6 months, less scar hyperplasia, and good evaluation satisfaction of all patients. No patient was rated as average or poor. No recurrence.

**Conclusion:**

The standardized surgical approaches for the small-size excision of melanocytic nevuses may be easily mastered by primary practitioners using the SF method, making it a valuable tool for practical use.

## Introduction

Melanocytic nevus is the most common benign skin tumor, which can be divided into intradermal nevus, mixed nevus and borderline nevus according to histopathology (1). Melanocytic nevus can occur in any part, especially in exposed areas such as face (2), which affects beauty, and the friction part is prone to canceration, which is harmful to health (3). Consequently, a realistic treatment strategy should be developed for melanocytic nevus based on its kind, size, location, indicators of malignant change, and the patients' motivation for receiving therapy (4).

When treating a small melanocytic nevus (≤1 cm), laser, freezing, electrocautery, or surgical excision are typically used. Nevertheless, it is challenging to remove pure melanocytes from pigmented nevi with deep bases using lasers (5, 6), cryotherapy (7), and electric burning (8). This results in a high recurrence rate and frequently leaves noticeable flaky scars at the laser treatment site.

We developed the SF (six cuts and five sutures) method for surgical resection of small-size melanocytic nevus through a large number of surgical summaries. We then compared it with normal method with the goal of proposing a standardized surgical method for resection of small-size melanocytic nevus that is easy for primary care physicians to master, has a short operation time, a simple operation method, and a good prognosis.

## Patients and methods

### Patients

This was a retrospective study. In accordance with the inclusion and exclusion criteria, 482 patients had skin melanocytic nevus resection from June 2022 to June 2023 at the Department of Plastic Surgery, Second Affiliated Hospital of South China University, and 241 patients had normal method (Table 1). The University of South China's Ethics Committee examined and approved the research methodology and treatment plan.

**Table 1:** General situation statistics of patients undergoing nevus removal.

Group	Number	Sex	Age	Area			Long diameter (cm)
		(Male/female, *n*)	(years old, x ± s)	Head and face, *n*	Torso, *n*	Four limbs, *n*	
SF method	482	256/226	41.3 ± 4.6	227	102	153	0.71 ± 0.27
Normal method	241	161/80	37.1 ± 5.2	135	67	39	0.64 ± 0.33

Selection criteria: (1) the length and diameter of skin melanocytic nevus ≤1 cm; (2) No laser, freezing, electrocautery, surgery and other traumatic therapy has been performed; (3) The age is 16–60 years old.

Exclusion criteria: (1) Patients with specific diseases like diabetes, coagulation dysfunction, or blood diseases; (2) Skin allergies, infections, or inflammations that occurred in the operation area within three months; (3) Smokers who smoked actively or passively within three months prior to and following the operation; and (4) Female patients’ menstrual cycles.

## Surgical procedure

Once the operation area has been marked, disinfect the wound and the 15 cm surrounding it with a complex iodine solution. Spread a sterile sheet and use the appropriate quantity of lidocaine solution (Hualu Pharmaceutical Co., Ltd. 5 ml:0.1 g) + epinephrine injection (Baiyunshan Mingxing Pharmaceutical Co., Ltd. 1 ml:1 mg) for local infiltration anesthesia.

### SF method

**Figure 1:**
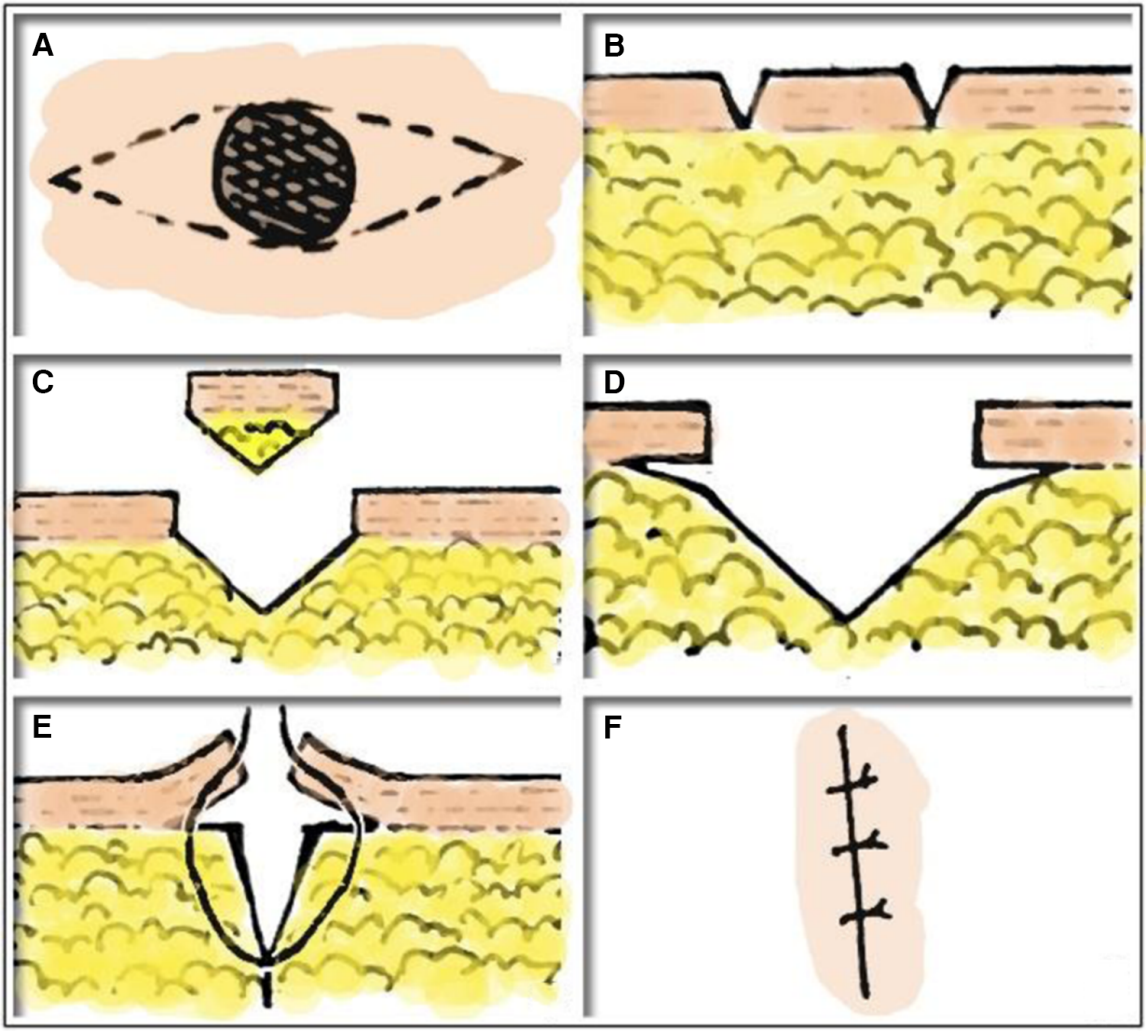
SF method. (**A, B**) Cut the edge of the melanocytic nevus perpendicular to the skin along the design line through the first two cuts to the subdermal skin. (**C**) And then cut the melanocytic nevus of the skin through two cuts in the subcutaneous tissue. (**D**) The skin and subcutaneous tissue were separated by making a transverse cut with a knife along both sides of the lesion. (**E**) Inversion suture using 5-0 absorbable thread was used to join the dermis on both sides and the soft tissue at the bottom of the incision. (**F**) 6-0 absorbable thread or 7-0 protein thread were chosen to close the wound.

1. Six cuts: cut the edge of the melanocytic nevus perpendicular to the skin along the design line through the first two cuts to the subdermal skin (Figures 1A,B), and then cut the melanocytic nevus of the skin through two cuts in the subcutaneous tissue (Figure 1C). At this time, a fusiform wound deep into subcutaneous tissue is formed. Ultimately, the skin and subcutaneous tissue were separated by making a transverse cut with a knife along both sides of the lesion (Figure 1D).
2. Five sutures: The incision was divided into three equal sections, and a two-needle inversion suture using 5-0 absorbable thread was used to join the dermis on both sides and the soft tissue at the bottom of the incision (Figure 1E). The skin began to fold spontaneously at this point. Ultimately, because the surgical sites were different, 6-0 absorbable thread or 7-0 protein thread were chosen to close the wound. This was accomplished by intermittently stitching three needles at the fourth equal position along the wound's lengthy diameter (Figure 1F).

Following the procedure, a tiny quantity of erythromycin gel (Huaqing Pharmaceutical Co., Ltd.) was applied, followed by a sterile dressing and pressure-bandaged.

### Normal method

The melanocytic nevus's morphological edge is completely excised, and the wound surface is then sutured immediately using matching sutures. Soft tissue folds are typically formed on both sides of the sutured wound during this procedure, and the look is uneven afterward. Some surgeons decide to keep stretching and trimming the skinfold, which causes the surgical incision to become excessively long in the end. Following surgery, a sterile dressing is applied, a little amount of erythromycin gel is applied, and the area is wrapped under pressure.

## Postoperative management

After the procedure, routine dressing changes and disinfections were performed every day, and depending on the patient's position, the stitches were taken out at different times. Following surgery, each patient received follow-up care for 1 and 6 months.

## Subjective assesses

Patients' operating times (min) were recorded, and a questionnaire was used to assess the surgical outcome satisfaction, complications, recurrence rate, and effectiveness. All patients were evaluated for wound scar characteristics using relevant scales by two doctors who did not participate in the study at a 3 and 6-month follow-up after surgery (9):

Vancouver Scar Scale (VSS) evaluates scars based on four indices: pigmentation, vascularity, pliability, and height/thickness, scoring from 0 to 15. A higher score indicates a more severe scar.
Visual Analog Scale (VAS) (10) rates scar characteristics such as vascularity, pigmentation, patient acceptance, observer comfort, and overall appearance. Scores from each category are summed up; higher scores indicate more severe scars.

Patient and Observer Scar Assessment Scale (POSAS) evaluates scar characteristics using two numeric scales completed separately by patients (PSAS) and observers (OSAS). Patient scores are influenced by pain, itching, pigmentation, thickness, confidence, and pliability. Observer scores consider capillary distribution, color, thickness, surface roughness, pliability, and scar location. Scores range from 1 to 10, with lower scores indicating scar areas closer to normal skin color and higher scores indicating more severe scars.

## Statistical analysis

Using SPSS 26.0 statistical software for data processing. Normally distributed continuous data are presented as mean ± standard deviation (x ± s), and between-group comparisons are conducted using independent samples *t*-test. Skewed distributed continuous data are presented as M (Q1, Q3), and between-group comparisons are conducted using independent samples Mann–Whitney *U* test. Count data are presented as numbers (%), and between-group comparisons are conducted using *x*^2^ test or Fisher's exact test. *p* < 0.05 indicates statistical significance.

## Results

For small size melanocytic nevus, the SF approach took an average of 5.60 ± 2.28 min to operate on, and after 3 months, there was no noticeable recurrence, making the outcome exceptional. All 482 patients were declared cured or effective. All patients expressed satisfaction in 100% of cases.

The average operation time of ordinary orthotopic resection was 8.32 ± 3.61 min, and all 241 patients were considered to be cured or effective. And there was no obvious recurrence after 3 months, and the effect was remarkable. 75.42% of patients were satisfied (Table 2).

**Table 2:** Comparison of postoperative general indexes between the two groups.

Group	Number	Operation times (min, x ± s)	Cure rate (%)	Recurrence rate (%)	Complication rate (%)	Satisfaction rate (%)
SF method	482	5.60 ± 2.28	100	0	0	100
Normal method	241	7.32 ± 3.61	100	0	0	75.42

At 3 and 6 months after surgery, the SF method group had lower VSS, VAS, OSAS and PSAS scores than the normal method group, and the differences were statistically significant (*p* < 0.05) (Table 3).

**Table 3:** Comparison of VSS, VAS, OSAS and PSAS score between 3 and 6 months.

Group	Number	VSS score		VAS score		OSAS score		PSAS score	
		3 months	6 months	3 months	6 months	3 months	6 months	3 months	6 months
SF method	482	6 (5,6)	4 (4,5)	3 (3,4)	2 (2,3)	15.7 ± 1.4	12.4 ± 2.7	14.7 ± 2.0	11.4 ± 2.5
Normal method	241	7 (6,7)	5 (4,6)	4 (3,5)	3 (2,4)	16.6 ± 2.1	13.6 ± 2.2	15.3 ± 2.6	13.4 ± 2.3
*z*		3.149	2.766	2.881	3.059	2.674	3.007	3.212	2.957
*p*		0.002	0.006	0.004	0.002	0.002	0.004	0.002	0.002

Compared with normal method, SF method has smaller scars and higher patient satisfaction.

**Figure 2:**
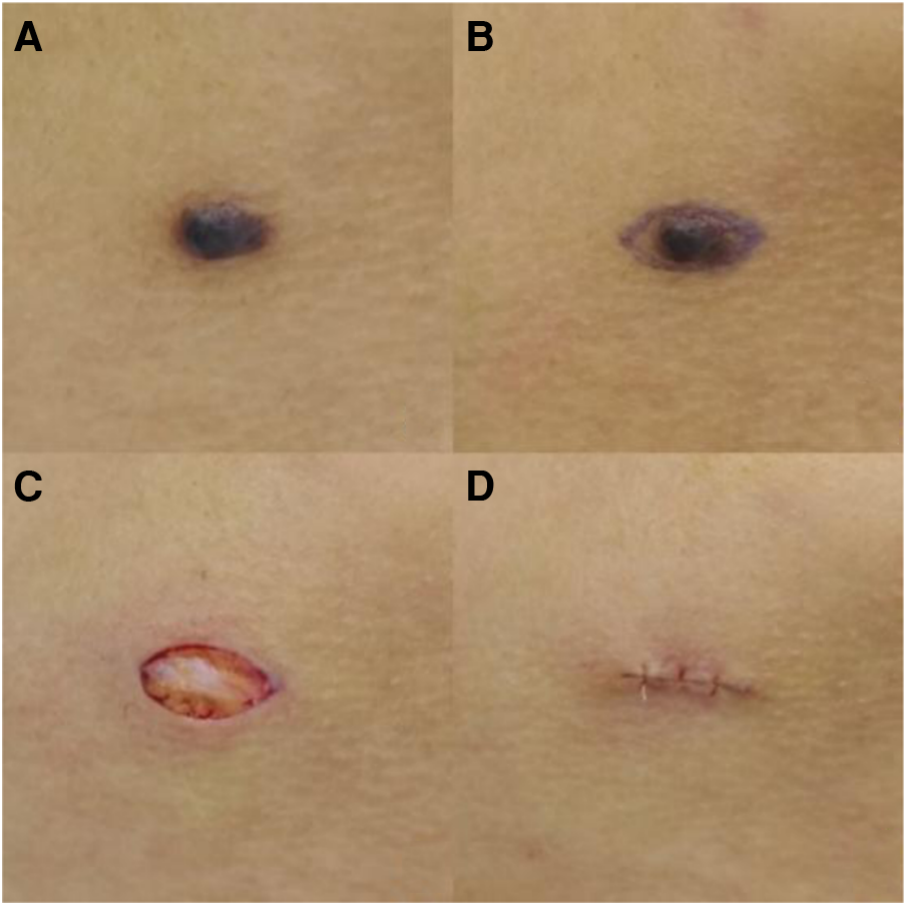
Typical case. (**A**) 0.6 cm long melanocytic nevus on the right mandibular surface, (**B**) preoperative incision, (**C, D**) resection by SF technique under local infiltration anesthesia.

A 17-year-old female patient, Figure 2A: 0.6 cm long melanocytic nevus on the right mandibular surface, Figure 2B: preoperative incision, Figures 2C,D: resection by SF technique under local infiltration anesthesia. There was no recurrence and no obvious complication 3 months after operation, and the patient were quite satisfied. After the procedure, the scar received a score of 1.

## Discussion

Pigmented nevus presents with distinct clinical and histological characteristics and is often referred to as congenital melanocyte nevus (2). It typically manifests at birth or soon after. Based on their size, congenital pigmented nevi fall into four categories: enormous, large, medium, and small nevi (11). Congenital melanocytic nevus affects about 2% of newborns; huge and giant nevus are uncommon. Small and medium-sized nevus make up the majority of these cases (12). Congenital pigmented nevi are often smooth, well-defined, black, round to oval, and regular. However, some pigmented nevi have the potential to develop into malignant melanoma (13).

Many people seek medical assistance to remove melanocytic nevi on the skin surface, especially those located on the face, driven by desires for aesthetic improvement or concerns about malignancy. Typically, we advise these patients to contact a dermatologist or plastic surgeon for assistance. Compared to general surgery, cosmetic suture technique (14) used in dermatology or plastic surgery can provide less visible scars and a more attractive appearance. Such low-level surgeries are typically carried out by junior physicians in many hospitals (15). It is therefore desperately essential to develop a quick, efficient, and elegant surgical technique for young physicians with limited experience. Thus, we summarized the resection of small melanocytic nevus into “SF” method by simplifying this flap technique, reducing the usage of surgical scissors, and using scalpels to operate the entire process, based on fusiform flap surgery in the field of classic plastic surgery (16).

In the “SF” method, a single skin melanocytic nevus is surgically removed in its entirety using six cuts and five sutures.

Six cuts: The first two are to cut the entire skin along the spindle-shaped design line, identify the tissue removal range, and split the skin from melanocytic nevus. In order to fully remove the skin-melanocytic nevus, the second cut in the skin incision should be made obliquely towards the inside. The final two incisions are made deep into the tissues on both sides of the wound, perpendicular to the initial two cutting lines, and release the skin tissues on both sides of the lesion. These incisions are made between skin and subcutaneous tissue, typically on the surface of fat or superficial fascia layer. Because skin is more elastic than subcutaneous tissue, this technique helps lessen wound strain following sutures (17). In this step, surgeons typically decide to employ scissors for blunt separation. Nevertheless, following numerous surgical procedures, it has been discovered that blunt separation using scissors not only lengthens the procedure but also provides superior skin and subcutaneous tissue protection and reduces hemorrhage. Long-term follow-up outcomes and our SF method do not differ in terms of prognosis; nevertheless, the direct use of blade sharp separation reduces operating time and improves patient satisfaction.

Five sutures: The skin's dermis layer and subcutaneous tissue are joined by the suture made by the first two needles. This suture method that lessens the wound's tension may also help to lessen scar hyperplasia. Ultimately, the skin epidermis on both sides of the wound can be aligned by three times intermittent sutures of the entire skin layer on the skin's surface, resulting in a smoother and smaller scar following wound healing.

This method is suitable for the removal of melanocytic nevi located on the surface of the skin. The only limitation is the location where the nevi exist. As long as there are no other structures nearby that affect the construction of the diamond incision, the SF method can be chosen without any limitations. In addition, this method can be used to treat medium-sized pigmented nevi, simply increasing the suture times as necessary. On the other hand, for large and giant nevi, this method can be applied gradually and removed after several operations, or it can be used directly for resection and repair using transfer flaps (18), expanded flaps (19), or even skin grafting (20).

## Limitations and strengths

The “SF” method presents both strengths and limitations in the context of surgical removal of melanocytic nevi. While it aims to minimize visible scarring, inherent risks of scar formation exist, particularly in cosmetically sensitive areas like the face. Its execution demands precise surgical skills for making six cuts and performing five sutures, potentially restricting its adoption to experienced practitioners. There's also a risk of recurrence or incomplete removal, especially with larger or complex lesions, and outcomes may vary widely among patients due to individual healing responses. Furthermore, the method's acceptance in the medical community may be hindered by a limited evidence base, lacking extensive long-term clinical studies and comparative trials against alternative techniques. Therefore, while offering potential cosmetic benefits, careful consideration of these factors is essential in its application.

The “SF” method offers several advantages in the surgical removal of melanocytic nevi. It prioritizes achieving superior cosmetic outcomes, particularly in sensitive areas like the face, by minimizing visible scarring. Its simplified technique, employing sharp blade separation instead of scissors, enhances surgical efficiency, potentially reducing operating time and enhancing patient satisfaction. Utilizing intermittent sutures, the method aims to diminish wound tension and scar hyperplasia, promoting smoother and smaller scars during the healing process. This approach is designed to be accessible to junior surgeons, providing a structured framework for achieving favorable cosmetic results even with limited experience. Ultimately, by enhancing patient comfort through minimized wound tension and superior aesthetic results, the “SF” method demonstrates promise in improving overall surgical outcomes for melanocytic nevi removal.

Overall, while the “SF” method shows promise in addressing aesthetic concerns and potentially improving surgical outcomes for removing melanocytic nevi, careful consideration of its limitations and appropriate patient selection is crucial for successful implementation.

## Conclusions

Compared with the normal method, this technique is simple, with good prognosis, less scar hyperplasia and easy for primary doctors to master, which is worthy of clinical application.

## Data Availability

The raw data supporting the conclusions of this article will be made available by the authors, without undue reservation.
